# Peanut-Induced Anaphylaxis in Children: A Literature Review

**DOI:** 10.7759/cureus.32946

**Published:** 2022-12-26

**Authors:** Hawra A Alshajarah, Hamza A Alghamdi, Zainab A Alberi, Fatima A AlAam, Abeer A Alshajarah, Maha F AlKhunaizi

**Affiliations:** 1 School of Medicine, Xi'an Jiaotong University, Xi'an, CHN; 2 Medicine, King Fahad Medical City, Riyadh, SAU; 3 Allergy and Immunology Section, Children Specialized Hospital, King Fahad Medical City, Riyadh, SAU; 4 Medicine, King Fahad University Hospital, Khobar, SAU; 5 Medicine, King Khalid University Hospital, Riyadh, SAU; 6 School of Nursing and Midwifery, Royal College of Surgeons in Ireland - Medical University of Bahrain, Muharraq, BHR

**Keywords:** emergency, epinephrine, children, anaphylaxis, peanut

## Abstract

Peanut allergy has become more common among children and is considered one of the most common triggers for fatal anaphylaxis. Treatment of symptoms during a reaction is only one aspect of managing anaphylaxis; other elements include rigorous dietary avoidance and education about settings that could put the patient at a high risk of unintentional exposure. We aimed to review the prevalence, mechanism, diagnosis, treatment, and emergency action of peanut-induced anaphylaxis among children. We used a web-based literature search using the advanced features of databases such as PubMed, Scopus, Directory of Open Access Journals (DOAJ), Embase, Google Scholar, and Cochrane electronic databases. The most common food to cause fatal anaphylaxis and a common cause of food allergies is peanuts. Over the past two years, our knowledge improved more about peanut allergens, their prevalence, causes, diagnoses, and treatments. The research cited in this review demonstrates that the peanut allergens are most closely associated with disease differ across cultures, that early oral peanut exposure may reduce the occurrence of peanut allergy while early non-oral exposure may have the opposite effect, that complement activation by peanut constituents appears to promote peanut-induced anaphylaxis, and that oral immunotherapy, anti-IgE antibody, and a herbal formulation are all demonstrating promise as treatments. To conclude, peanut allergies have increased frequently during the past 10 years, especially in Westernized nations. Given that peanut allergy poses a danger for fatal anaphylaxis, response management is crucial. The current standard of care for those with nut allergies comprises complete food avoidance and the administration of injectable epinephrine to treat systemic symptoms.

## Introduction and background

Introduction

Anaphylaxis is a severe, multisystemic, potentially life-threatening allergic reaction [1]. Around half of the reported cases of anaphylaxis are due to food allergies. In children, food is one of the most common causes of anaphylaxis in outpatient settings. Among all causative foods, peanuts, tree nuts, fish, and shellfish are responsible for most cases of severe anaphylaxis. Peanut allergy cases are common as peanuts are cheap, widely consumed in their natural state, and used as a component in a wide variety of prepared foods [2-5].

Peanut allergy (PA) is an abrupt and sometimes fatal allergic reaction that affects many bodily organ systems and occurs after the ingestion of peanuts [6]. It is estimated that around 0.6% of people in the United States have a PA [7-8]. Rates range from 1.5% to 3% among children in the West and have gone up over time [9-10]. As low as 50 µg of peanuts can initiate an allergic reaction which can occur either immediately after the ingestion, within hours after intake, or after days from the ingestion [11-12].

After exposure to peanuts, such as after eating peanuts or tree nuts, mast cells and basophils release mediators that contribute to the pathophysiology of anaphylaxis [5]. Pathophysiologic activities brought on by the release of mediators, including histamine, leukotrienes, and prostaglandins, smooth muscle contraction, increased vascular permeability, vasodilation, and stimulation of the nervous system with reflex vagal activation [6].

Injectable epinephrine remains the gold standard of care to treat anaphylactic shock including peanut-induced anaphylactic shock. In addition, oral immunotherapy (OIT) treatments have shown good effectiveness and safety in managing PA. Nonetheless, these treatments are not widely used in allergy clinics [5-13]. As there are many scenarios in which difficulties could occur if not adequately foreseen, counseling and educating the patient and family are time-consuming [14]. Herein we aimed to review the prevalence, mechanism, diagnosis, treatment, and emergency action of peanut anaphylaxis among children.

Materials and methods

A web-based literature search was conducted using several databases, including PubMed, the Directory of Open Access Journals (DOAJ), Embase, Google Scholar, and the Cochrane electronic databases. The databases were searched using the primary MeSH and other keywords such as Etiology, Diagnosis, Treatment, Quality of life, and so on. The search covered the most recent full-text review articles published between 2000 and 2022 and was limited to English articles. We chose 234 articles, and after removing duplicates, we were left with 65 articles for the review’s final research. Additional material was searched from the reference lists of the included studies. Studies conducted in a language other than English were not considered.

## Review

Prevalence

Because different definitions of anaphylaxis are employed, data on the frequency of anaphylaxis in patients with PA are scarce. According to numerous research conducted in Western countries, severe allergic reactions to peanuts happen more frequently than allergic reactions to other foods [15-16]. Children with PA were more likely to have a history of severe responses than children with other food allergies (42.3% rate of severe reactions overall), according to a US study on food allergies in children (n = 38,408) [15]. Similar to this, a nationwide 2009-2010 US survey study including 38,480 children discovered that considerably more children with PA (n = 754) than children with food allergies in general (n = 2464) had severe reactions to peanuts (53.7% vs. 41.0%; p = 0.001) [16]. In contrast to the 51% overall rate of any severe food-allergic reactions among all US adults with food allergy, 68% of US adults with PA report at least one severe peanut-allergic reaction [17]. The Australian School Nuts Study, which examined 547 adolescents between the ages of 10 and 14 who may have had food allergies, discovered that reactions to peanuts accounted for the highest percentages of any food sources, accounting for 38.6% of confirmed anaphylaxis episodes and 30.6% of unconfirmed attacks [18].

A UK clinical practice database study found a significantly lower anaphylaxis rate of 1.2% of all patients (children and adults) with PA vs. 0.007% of matched controls [19]. A cross-sectional nationwide US study of 754 children with PA reported an anaphylaxis rate of 14.2%, compared with 8.1% in children with other food allergies. According to another US study, 35% of 525 children experienced anaphylaxis over five years [20]. Children with mild initial reactions that led to diagnosis commonly experience accidental exposures that result in anaphylaxis, illustrating the unpredictability of PA reaction intensity [21-23]. Although it is challenging to estimate the frequency of anaphylaxis to peanuts due to different reporting procedures and anaphylaxis definitions, it is clear that individuals with PA frequently experience severe and unintentional reactions. Additionally, several studies used self-report, susceptible to recall bias and misclassification. There is a need for more research on the most recent, reliable, and validated diagnostic criteria for anaphylaxis [24].

Pathophysiology

In peanuts, there are different allergenic proteins abbreviated as Ara h1 to Ara h11; *Arachis hypogaea* 1-11. These proteins are important in the pathogenesis of peanut anaphylaxis through the provocation of IgE-mediated reactions. The pathogenesis of peanut anaphylaxis begins post the exposure to allergenic proteins in susceptible individuals [25].

Once in the body, these allergenic proteins are recognized as foreign bodies, phagocytosed by antigen-presenting cells (APCs) such as dendritic cells and macrophages, and then will be presented to T-cells in the lymph nodes. The function of APCs is driven by pro-inflammatory cytokines like interleukin 25 (IL-25) and IL-33. Thereafter, T cells will differentiate into T-helper cells through the effect of the MHC class II receptor and IL-4. After that, T-helper cells will differentiate into the TH2 subtype [26].

TH-2 cells release IL-5 which helps in eosinophil function, IL4 which promotes class switching of B-cells and IL-9 which regulates mast cell activation. TH-2 cells also stimulate the activation of B-cell to become plasma cells through IL-12. In addition, T-helper cells also secrete IL-4 & IL-13 which leads to class switching of B-cells to plasma cells and result in the formation of antigen-specific IgE antibodies. Plasma cells then will secrete specific antibodies to the peanut allergens. The antigen-specific antibodies (IgE) bind to the IgE receptors on mast cells and basophils. This phase is known as the sensitization phase [26].

In subsequent exposures to the peanut allergens, peanut antigens bind to IgE antibodies through specific epitopes, crosslinking of FcεRI-bound occurs in the mast cells and basophils, mast cells activation occurs and inflammatory mediators get released, including histamine, prostaglandins, and tryptase. The release of these mediators results in the clinical features of peanut anaphylaxis. Release of histamine results in vasodilation and pruritus. In addition, serum tryptase can help in the diagnosis of anaphylaxis.

Among all peanut allergenic proteins, Ara h 2 proteins have a more stable intrinsic structure due to the disulfide bonds and cysteine residues. This allows these proteins to cross the mucosal layer in the gut and sensitize the immune system. Ara h 2 proteins belong to the 2S albumins group which are characterized by high allergenicity and cross-reactivity of different foods [27].

Diagnostics for peanut allergies

Even when a patient may not have a clinically severe allergy to the target allergen, specific IgE immunoassays (sIgE) (see above) may produce a positive result. The sIgE approach employs extract from the tree or peanut nuts. IgE in some proteins is more strongly related to severe allergy than other proteins, and not all peanut and tree nut proteins are equally allergenic. Since Ara h 8 is analogous to Bet v one and other birch pollen allergens and hence associated with the milder oral allergy syndrome, people with sIgE to the allergenic protein Ara h 2 are more likely to experience severe, systemic reactions to peanuts than patients with Ara h 8 [28]. Recombinant protein immunoassays, instead of extracts, are used in component-resolved diagnostics (CRD) to identify the specific proteins to which patient antibodies are reactive.

When a patient has a positive sIgE test for peanuts, CRD can reveal which of the Ara proteins the patient’s antibodies are reactive to and, as a result, predict how severe their allergy will manifest clinically. Early research suggests that CRD is an effective method for making these forecasts [29]. Less than half of patients with peanut allergies who avoid tree nuts are likely to also have tree nut allergies, according to the findings of a recent trial using CRD in 108 patients with peanut allergies. This finding could be attributed to the cross-reactivity among these foods. The clinical significance of these findings has not yet been confirmed [30] since the tree nuts examined for this investigation were not subjected to an oral feeding challenge (OFC) test.

In general, evaluation for PA with sIgE levels is recommended only in patients with high pretest probability or prior to the oral food challenge test. In addition, assessing Ara h 2 provides the best diagnostic and prognostic accuracy. The classic “skin prick test” which involves administration of the diagnostic allergen in the outpatient settings and assessing the skin’s response remains the gold-standard test for PA. Other diagnostic tests include basophil activation test and peanut bead-based epitope assay are new diagnostic tests that need further assessment and study. However, the diagnosis of PA can be challenging as sensitization is not equal to clinical allergy. Sensitization can also occur due to cross-reactions to other allergens like grass pollen. Furthermore, low levels of peanut proteins IgE do not exclude the presence of PA which can be seen in up to 25% of the cases [31-32].

Anaphylaxis emergency action (EAs) plans and medical identification

The possibility of a life-threatening reaction may be quite natural for doctors and families of people with peanut allergies. Naturally, not every food reaction is severe. For all foods, including peanuts, the rate of responses necessitating emergency department (ED) care appears modest [33]. The ideal way to prescribe EAs is within a written, individualized anaphylactic emergency action plan. These plans often identify the typical anaphylactic symptoms and warning signals and specify the initial anaphylaxis treatment, which emphasizes phoning for assistance (911 or EMS), administering epinephrine from an EA, and putting the patient supine or in a comfortable posture [33-36]. Action plans can include details about a person’s anaphylactic triggers, history of severe anaphylaxis, or any comorbid diseases like asthma, if applicable. They can also remind readers that anaphylaxis should not be treated with H1-antihistamines or asthma inhalers as the first or only course of action [36].

Patients who are in danger of experiencing anaphylaxis again can carry a wallet card that says “anaphylaxis” and specifies their proven triggers as well as any pertinent comorbidities, such as asthma, and wear medical identity jewelers. After a quick review of an allergy wallet card, knowledge of the diagnosis and treatment of anaphylaxis significantly increased [37]. It is best to review and update medical IDs and plans regularly, such as once a year [7,34-35].

This was demonstrated by Capucilli et al. in a sizable pediatric cohort, where the incidence rate of ED visits due to an unintentional food response was only 0.5% [38].

An increase in hospital admissions and visits to EDs is also consistent with an increase in the use of healthcare services for food-induced anaphylaxis (FIA) [39-41]. The number of ED visits due to FIA for children under 18 did not statistically differ between 2001 and 2009 [42].

Emergency kit

An auto-injector for epinephrine, a histamine H1 receptor antagonist, a glucocorticoid, and, in the event of bronchial asthma or a history of bronchospasm, an inhaled bronchodilator (2-adrenoceptor agonist), if necessary, with the proper inhalation assist, should all be included in the emergency kit. It is mainly prescribed for those at high risk of anaphylaxis. 150 mcg, 300 mcg, and 500 mcg are the available doses used in anaphylaxis. An inhaled epinephrine formulation may be considered if there has been a history of significant laryngeal edema. With every emergency pack, an anaphylaxis action plan or passport should be included [43].

Treatment of peanut anaphylaxis

The most crucial medication for anaphylaxis is adrenaline [44-45]. Its use to lessen bronchoconstriction and restore appropriate tissue oxygenation is supported by observational data, clinical experience, and animal models, even though there are no randomized controlled trials. Adrenaline reverses peripheral vasodilation and lessens tissue edema as an alpha-receptor agonist. The myocardial contraction force increases, the bronchial airways are dilated, and histamine and leukotriene production are suppressed by its beta-receptor activation. Early adrenaline may lessen the severity of IgE-mediated allergy reactions because it directly inhibits the activation of beta-2 adrenergic receptors on mast cells [46-48]. When administered soon after the onset of anaphylactic symptoms, adrenaline appears to operate best [48]. Delayed administration is linked to catastrophic results, hypotension, and prolonged reactions [49]. Adrenaline is not without risk, especially when administered intravenously [45]. With the proper doses given by the IM method, adverse effects are infrequent. Rarely have problems been recorded (such as myocardial ischemia). However, it’s unclear if they were brought on by the allergic reaction, the adrenaline used to treat it, or a combination of the two. If the clinical picture changes when the patient is being evaluated, problems may occur. All patients with life-threatening symptoms (i.e., indications of airway/breathing/circulatory involvement) should be given adrenaline. The patient requires careful observation and appropriate symptomatic treatment utilizing the ABCDE strategy if these symptoms are absent, but there are still additional features of a systemic allergic reaction. Where anaphylaxis might happen in a clinical setting, adrenaline must be easily accessible. This includes locations outside hospitals, for example, where immunizations may be given [50].

The anterolateral side of the middle part of the thigh is the ideal location for IM injection [51]. Use a green (21G) or blue (23G) needle if the injection needle needs to be long enough to inject the adrenaline into the muscle [52]. Because they are less effective, administering adrenaline subcutaneously or inhalation is not advised for anaphylaxis [51-54].

IM adrenaline is considered as it is safer than IV adrenaline (Table 1). The recommended doses are not supported by strong scientific evidence. According to international guidelines, the suggested amounts are based on what is deemed safe and practicable to draw up and inject in an emergency [55]. If the patient’s condition does not improve after 5 minutes, repeat the IM adrenaline dose. Although the evidence is unclear, some recommendations urge additional dosages in the opposite thigh to help with absorption. The reaction to adrenaline varies significantly between people, with peak absorption happening 5 to 10 minutes after IM injection [55]. Attaching the patient to a cardiac monitor (pulse oximetry, blood pressure, and electrodes leas) will help assess for any improvements and respond to adrenaline doses. Follow the refractory anaphylaxis algorithm if there is no improvement in breathing or circulation issues despite two amounts of adrenaline [55].

**Table 1:** Dose of IM adrenaline to control anaphylaxis [55] *Give 300 µg IM (0.3 mL) to a child who is small or prepubertal

Adrenaline IM dose	Use 1 mg/mL (1:1000) adrenaline	
Adult and child* > 12 years	500 µg IM	(0.5 mL of 1 mg/mL adrenaline)
6–12 years	300 µg IM	(0.3 mL)
6 months–6 years:	150 µg IM	(0.15 mL)
< 6 months:	100–150 µg IM	(0.1–0.15 mL)

After receiving 2-3 doses of adrenaline, children may still be pale [56]. An important symptom of an adrenaline overdose is markedly elevated blood pressure [57]. To evaluate the effectiveness of the treatment and determine whether additional doses of adrenaline are necessary, measure vital signs (respiratory rate, oxygen saturation, heart rate, blood pressure, level of consciousness) and auscultate for wheezing [55].

Due to dilution mistakes or improper doses, there is a significantly increased risk of adverse side effects when administering IV adrenaline [58-61]. Adrenaline overdoses, especially those administered via IV, can result in tachyarrhythmias, severe hypertension, myocardial infarction, and stroke. The improper use of IV adrenaline to treat allergic reactions other than anaphylaxis has resulted in fatalities in the UK. Adverse effects are more prevalent following IV adrenaline delivery, especially with IV bolus administration and dosing errors (e.g., using 1 mg/mL (1:1000) solution (suitable for IM injection) instead of more dilute solutions (e.g., 0.1 mg/mL (1:10000) for IV injections) [34].

Nebulized adrenaline may be helpful as a supplement to treat upper airway blockage brought on by laryngeal edema, but only as a follow-up to treatment with IM (or IV) adrenaline. 3-5 mL of 1 mg/mL (1:1 000) adrenaline are advised doses that may cause minor side effects. To obtain 4-4.5 mL in total volume, it is mainly combined with 0.9% sodium chloride [62].

The highest possible oxygen concentration should be administered initially using a mask with an oxygen reservoir (more than 10 L/min). Consider a target range of 88-92% in individuals at risk for hypercapnic respiratory failure. Change the inspired oxygen concentration as soon as possible to reach an oxygen saturation of 94-98%. Intubated patients will have high concentrations of oxygen ventilated into their lungs [45].

Antihistamines’ involvement in anaphylaxis is controversial, although it is agreed upon by all guidelines that they should not be used as the first line of treatment [44-45,57,62]. Treatment of anaphylaxis’ life-threatening symptoms does not benefit them. Most recommendations indicate they worry that their frequent use prolongs the time between administering the initial and subsequent doses of adrenaline, increasing morbidity [63]. Although antihistamines can aid with cutaneous symptoms, they shouldn’t be used in place of adrenaline to treat anaphylaxis [56].

Corticosteroids’ main effect suppresses the late-phase (as opposed to early-phase) inflammatory response. However, the effectiveness of corticosteroids in reducing protracted symptoms or avoiding biphasic reactions is not well supported (see section 8.2) [63-64]. Even after controlling for the severity of the presenting symptoms, accumulating data imply that early steroid usage is linked to an elevated risk of intensive care admission [65-66]. These suggestions for steroid use are supported by evidence with a lesser degree of assurance [63].

Consider further inhaled bronchodilator therapy with salbutamol or ipratropium in addition to the medications on the preceding list. There is no evidence to suggest that one bronchodilator should be preferred over another when treating anaphylaxis [45]. If respiratory issues persist, bronchodilators shouldn’t be utilized as a substitute for additional parenteral treatment with adrenaline. Adrenaline is the primary drug treatment for anaphylaxis [56].

Through the production of regulatory T-cells, Liu et al.’s liver-targeting nanoparticle platform can treat allergic inflammation, mast cell release, and anaphylaxis (Treg) [67]. By encapsulating and delivering the predominant protein allergen Ara h2 and representative T-cell epitopes to liver sinusoidal endothelial cells, they show how to use a poly (lactide-co-glycolide acid) (PLGA) nanoparticle platform to treat peanut anaphylaxis (liver sinusoidal endothelial cells (LSECs)). These cells have the ability to function as natural tolerogenic APCs, generating Tregs by presenting T-cell epitopes to LSECs via MHC type II complexes. This made it possible to address the theory that an efficient, secure, and scalable intervention for reducing allergy to crude peanut allergen extract might be made using the tolerogenic nanoparticle platform. The best-performing Ara h2 T-cell epitopes were compared with purified Ara h2 allergens, a crude peanut protein extract (CPPE), and a control peptide in an oral sensitization paradigm after the investigation of isolated Ara h2 and representative MHC-II epitopes for Treg production in vivo. In a commonly used PA paradigm, mast cell protease release, hypothermia, and anaphylactic symptoms were all eliminated more effectively with the dominant encapsulated Ara h2 T-cell epitopes than with the pure Ara h2. TGF release in the abdominal cavity rose, while blood levels of peanut-specific IgE dropped. The preventive effect persisted for two months. These findings show that a successful platform for treating PA anaphylaxis could be created by carefully focused delivery of T-cell epitopes to naturally tolerogenic liver APC.

Other treatment options, such as multiple OIT under concurrent omalizumab therapy, the simultaneous use of OIT and dupilumab, or hypoallergenic peanut extract for subcutaneous administration, as well as oral mucosal immunotherapy (OMIT) via toothpaste peanut protein administration, are being researched in clinical trials. The clinical phase of peanut immunization using the virus-like particle platform is almost complete. The effectiveness of OIT in this age range for endpoint desensitization was recently demonstrated in a double-blind placebo-controlled trial in the USA with 146 1 to 3-year-old peanut-allergic children [68]. Only one patient (2% of the placebo group) and 71% of the verum group were able to tolerate 5 g of peanut protein during oral challenge testing after 134 weeks of OIT. A sustained inability to respond to 5 g of peanut protein was found in 21% of the active group and only in one patient (2%) of the placebo group after a treatment break of 6.5 months [68]. In phase-III trials in children aged 1-3 years, OIT with AR101 and epicutaneous immunotherapy are both being studied. As a result, people with peanut allergies of all ages will likely have access to various therapeutic choices in the coming years.

## Conclusions

PAs have increased frequently during the past 10 years, especially in Westernized nations. Nut allergy is dangerous for fatal anaphylaxis, so response management is crucial. The current standard of care for those with nut allergies comprises complete food avoidance and the administration of injectable epinephrine to treat systemic symptoms. The dietary release can be challenging, particularly for young children and in unique settings like restaurants where the potential for cross-contamination is still very high. Although there is still much need for improvement in education and awareness, labeling processed foods and community knowledge of nut allergies have helped to make dietary avoidance slightly easier. Given the impact food allergies have on the quality of life, it is not unexpected that research into alternative and more effective therapies for people with peanut allergies has received a lot of attention.
